# Play with Your Food and Cook It! Tactile Play with Fish as a Way of Promoting Acceptance of Fish in 11- to 13-Year-Old Children in a School Setting—A Qualitative Study

**DOI:** 10.3390/nu12103180

**Published:** 2020-10-17

**Authors:** Rikke Højer, Karen Wistoft, Michael Bom Frøst

**Affiliations:** 1 Center for Nutrition and Rehabilitation, Nutrition and Health, University College Absalon, Slagelsevej 70-74, 4180 Sorø, Denmark; 2 Department of Food Science, Design and Consumer Behaviour, University of Copenhagen, Rolighedsvej 26, 1958 Frederiksberg, Denmark; mbf@food.ku.dk; 3 Department of Educational Sociology Emdrup, Danish School of Education, Tuborgvej 164, Building D, 143, 2400 Copenhagen, Denmark; kawi@edu.au.dk

**Keywords:** food acceptance, tactile play, cooking, children, fish, health promotion

## Abstract

Despite a tradition of consuming fish in Denmark and despite the health benefits of eating fish, Danish children consume only one-third of the officially recommended amount of fish. The objective of this study was to explore an experiential and sensory-based exercise in a school setting with focus on tactile play and cooking as a way of promoting 11- to 13-year-old children’s acceptance of fish. The design was a qualitative exploratory multiple-case design using participant observation in a school setting. Six classes were recruited from the Eastern part of Denmark (*n* = 132). Based on an exercise with cooking fish and gyotaku (fish print), four meta-themes were identified by applying applied thematic analysis: rejection, acceptance, craftsmanship, and interaction. Rejection and acceptance appeared along a rejection–acceptance continuum related to how the fish was categorised (animal, non-animal, food) in different phases of the experiment. Rejection was promoted by mucus, smell, animalness, and texture, whereas helping each other, tactile play, and craftsmanship promoted acceptance. In conclusion, this study found that tactile play combined with cooking could be a way of promoting acceptance of fish. The findings also support a school setting as a potential gateway in promoting healthy food behaviour.

## 1. Introduction

### 1.1. Background

Children aged 11 to 13 years are in the early adolescent life phase [1], a phase defined by a developmental plasticity [2], where lifelong habits can be established [3]. The adolescent life phase is critical when it comes to behavioural changes in, for example, dietary habits [4]. The changes in dietary habits are due to, for example, an increase in autonomy and a decrease in family influence [5,6].

Consumption of fish provides valuable nutrients. Especially fatty fish have a high content of vitamin D, which is important for e.g., calcium (Ca) absorption, bone health, and childhood growth stages [7,8]. Regular consumption of fish, especially those high in n-3 poly unsaturated fatty acids (PUFA), also reduce incidences of, for example, diabetes mellitus, systemic arterial hypertension, central obesity and hyper-lipidemia [9,10], and seem to positively influence intestinal microbiota [11]. Furthermore, the macro nutrient content of fish with regard to protein is 15–20% and fish contains all the essential amino acids [12], which is beneficial for the diet as the sulphur-containing amino acids, cysteine and methionine, are absent in plant protein. Furthermore, proteins from fish have a high degree of digestibility i.e., 85–95% [12,13]. Studies have shown positive health effects as a result of fish protein intake e.g., by decreasing the risk of metabolic syndromes and increasing insulin sensitivity [14,15,16,17].

#### 1.1.1. Acceptance and Rejection of Food

This study focuses on fish as part of a healthy diet. According to a national study, Danish children aged 10 to 17 years eat only 105 g of fish per week [18], one-third of the Nordic recommendations of 350 g per week [19]. The intake of fish among Danish early adolescent phase children corresponds with international observations [20,21,22]. Furthermore, to the authors’ knowledge, little research has been conducted in the area of early adolescent phase children’s acceptance of fish.

Rozin and Fallon [23,24] have developed a framework in which they have identified three principal motivations within the taxonomy of food acceptance and rejection, which drive food acceptance and rejection: sensory-affective factors (e.g., liking/disliking taste or smell), anticipated consequences (e.g., negative/positive physiological or social), and ideational factors (e.g., knowledge of the nature or origin of a food). These motivations and attributes can lead to either rejection or acceptance: the psychological rejection categories are distaste (the concept distaste includes all sensory characteristics, real or imagined [25,26]), danger, inappropriateness, and disgust, and the acceptance categories are good taste, beneficial, appropriate, and transvalued [25,27]. Furthermore, Rozin and Vollmecke [27] point out that the influence of culture and context are predominant factors influencing acceptance and rejection, and that acquired likes can be promoted by social encounters with people outside the family, especially peers. The framework of rejection and acceptance developed by Rozin and colleagues [23,24,27] has been applied repeatedly in studies investigating food behaviour (e.g., [25,26,28,29]).

Based on the limited research conducted within and around the target group of this study, Prell, Berg, and Jonsson [30] identified a negative attitude towards the smell, the fear of finding bones, the accompaniments, and friends’ behaviour as primary barriers to eating fish. In a study focusing on foods in general, Frerichs et al. [31] found that appearance and texture were primary drivers for accepting or rejecting food. Furthermore, Mitterer-Daltoé, Latorres, Treptow, Pastous-Madureiraa, and Queiroz [32] and Latorres, Mitterer-Daltoé, and Queiroz [33] found that young children had a higher acceptance of fish than older children. This might be due to the older children’s cognitive maturation, leading to food-related cognitions increasing and becoming more complex [34]. The animal origin of fish could also play a role in rejection, since foods of animal origin tend to promote an attitude of disgust more than those of vegetable origin [24,25,35,36]. Increasing acceptance of food through tactility (the sense of touch by using the hands) or tactile play is a research area that has yet to be explored in greater depth. Five recent studies have been conducted in this research area [37,38,39,40,41,42], but these studies all fall outside the age-related sample of this study. Nevertheless, the results are interesting and relevant to this study as they point to a positive impact of tactile play on food neophobia and/or food acceptance.

Another way of influencing food behaviour and promoting acceptance of healthy foods has been sought through a hands-on approach and cooking programmes. A review of the effect of cooking programmes by Utter, Fay, and Denny [43] concluded that cooking programmes may have a positive impact on food-related beliefs, knowledge, skills, and behaviours. Of the 20 studies included in the review, only three were on children in the age range of the sample group in the present study. However, none of the studies included in the review focused on foods of animal origin. Furthermore, observations of children’s food behaviour and learning processes have been included in studies by, for example, Block et al. [44], Fisher and Birch [45], and Gibbs et al. [46]. The relevance of applying observation as a research method relates to the objective of revealing actual behaviour.

**Figure 1:**
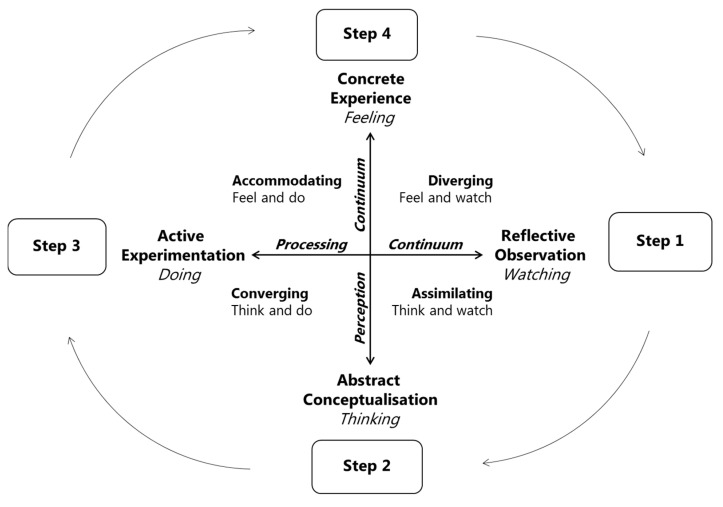
Model of culinary skills education as a process for Kolb’s cycle of experiential learning developed by Nelson, Corbin, and Nickols-Richardsson [47]. Figure by first author R. Højer.

Nelson, Corbin, and Nickols-Richardsson [47] argue that culinary skills education offers a unique opportunity for experiential learning, which they illustrated through the use of the Kolb Cycle of Experiential Learning [48] combined with culinary skills education (Figure 1).

According to Nelson et al. [47], culinary skills education promotes knowledge through experience, as illustrated in Figure 1. As students move from observational to experiential learning stages and engage in culinary concepts, a foundation for promoting critical thinking and learning skills and technical proficiencies is laid out, all aimed at promoting healthy food behaviour. Furthermore, Nelson et al. [47] conclude that nutrition knowledge alone, aimed at promoting healthy food behaviour, seems incomplete without the dimension of experiential learning via interactions with food and cooking equipment.

#### 1.1.2. The Subject Food Knowledge

In 2014, the subject Food Knowledge replaced the subject Home Economics as part of a reform of the Danish compulsory primary and lower secondary schools. The subject is mandatory for one year and can be taken in 4th, 5th, or 6th grade. In the subject Food Knowledge, students focus on four areas of competencies: Food and Health, Knowledge of Food, Cooking and Dining, and Food Cultures. The purpose of the reform was to ensure that Food Knowledge provides students with an opportunity to work with senses and experiences. Experimentation, creation, and communication in relation to food and meals are also key elements, as is the development of, for example, new skills and knowledge through motor skills, cognition, and perception [49].

#### 1.1.3. Gyotaku Explained

**Figure 2:**
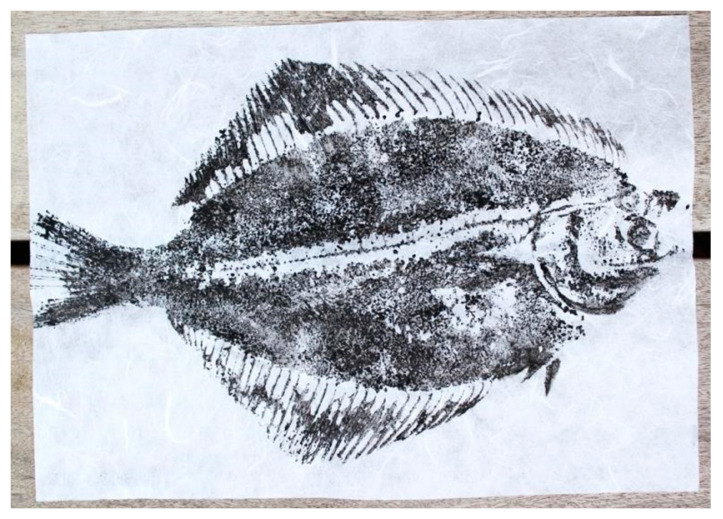
Gyotaku of flounder *(Platichthys flesus)*, artist: R. Højer, photo: Marilyn Koitnurm.

Gyotaku is a traditional Japanese art form (see Figure 2); gyo is the Japanese for fish and taku for rubbing or printing: fish rubbing or fish printing [50].

Gyotaku was used by Japanese fishermen more than a hundred years ago [51]. To avoid misunderstanding, the fishermen used it to replicate the correct size of the fish, whereby it became a documentation method. During the twentieth century, the practice of gyotaku has been turned into an art form.

As an example of an experiential exercise, gyotaku was adapted to firstly include a tactile art exercise, which was the traditional part of the exercise to be explored in this study. Secondly, after the art part of the exercise in which the fish served as an art medium, the fish would then be included in a cooking exercise. The gyotaku exercise was chosen for its novelty in a Danish context and for its tactile hands on approach to the fish.

### 1.2. Study Aim

The aim of this study was to promote children’s acceptance of fish. Based on the hypothesis that through hands on experience with fish it is possible to promote acceptance of fish, the objective of this study was, through an intervention, to explore the potential of a sensory-based experiential exercise in a school setting with focus on cooking and tactile play as a way of promoting 11- to 13-year-old children’s acceptance of fish. The two main research questions to be answered were: (1) how do children respond to handling, preparing and cooking fresh fish? and (2) how does the process of the sensory-based tactile experiment gyotaku affect children’s acceptance of fish? 

## 2. Materials and Methods 

### 2.1. Study Design

This study was an intervention with a multiple-case study design [52]. Six cases in six different classes from six different schools were included in the intervention. All participating classes underwent the experiential gyotaku exercise one class at a time. The qualitative method used to collect data consisted of participant observation [53]. 

The gyotaku exercise was integrated into the (in Denmark) compulsory subject Food Knowledge (*Danish: Madkundskab*) [49] in the fifth and sixth grades and it meets the official learning goals (for 2017–2018 and 2019) for the subject Food Knowledge set by the Ministry of Children and Education [54].

#### Ethics Approval

Ethics approval for this study was given by the joint Research Ethics Committee of the Faculty of Science and the Faculty of Health and Medical Sciences, University of Copenhagen, Denmark (reference 504-0005/17-5000).

### 2.2. Participants

We recruited six classes from fifth and sixth grades (11 to 13 years of age) from six different Danish public schools (*n* = 132). Four classes were from the capital region and two from the region of Zealand (see Table 1 for participant characteristics). Recruitment was geographically limited to the eastern part of Denmark due to convenience. The recruitment was done by sending out information letters via e-mail to schools in the eastern part of Denmark addressed to the school’s Food Knowledge teachers. For all participating children, written informed consent was given by the legally appointed caregiver parent or either parent if the parents were married or had joint custody. Children’s refusal to touch, handle, and/or taste the fish was respected by the researchers. 

### 2.3. Setting and Gyotaku Exercise

School SA, SB, and SC took part in gyotaku workshops in a teaching kitchen at the Department of Food Science at the University of Copenhagen, Frederiksberg, Denmark, in a field trip setting. School MB, MC, and MD were in their natural educational setting, since the gyotaku exercise took place on three different occasions at schools in the ordinary school teaching kitchen. This differentiated setup was due to practical organization as the classes SA, SB and SC participated as part of Science Week 2016, a yearly returning science festival in Denmark, whereas the classes MB, MC, and MD did not participate in Science Week 2016 and data were collected during early spring 2017. All classes carried out the gyotaku exercises based on the same exercise guide.

The sensory-based experiential exercise was a four-phase exercise consisting of a) gyotaku (fish printing), which also gave its name to the complete experiment, b) filleting a fish, c) cooking the fish fillets by a commonly used Danish method, and d) tasting.

Materials for the gyotaku experiment (per group of four children): one fresh whole flatfish with head (either dab (*Limanda limanda*) or flounder (*Platichthys flesus*)), one lemon, squid ink diluted with tap water in a cup, a small sponge, five A4 pieces of paper cut into eight equal parts, paper towels, printing paper, a cutting board, a sharp filleting knife, rye flour, salt, pepper, butter, rye bread, a frying pan, a stove and written experimental instructions. 

General organisation: all of the children worked in groups of four. Each group received one fresh fish to be shared during printing, filleting, and cooking (1 fish = 4 fillets).

Phase a: Gyotaku (printing): The printing procedure was the actual gyotaku exercise. The children chose and picked up their group’s fish from a box containing fresh fish on ice. The fish was then cleaned by washing it under cold running water while rubbing it with a slice of fresh lemon (this dissolves the fish’s natural mucus cover). The fish was then dried with paper towels and placed on a cutting board. Paper squares were placed around the edge of the fish to avoid getting squid ink on the cutting board. Diluted squid ink was applied with a sponge to the surface of the fish until it was covered with ink. The paper squares around the fish were removed, and printing paper was placed on top of the fish. The print was transferred to the paper by stroking the fish on top of the paper. The paper was gently pulled off the fish, and a mirrored print of the fish had been transferred to the paper (see Figure 2).

Phase b: Filleting: If they wanted to, each child in the group filleted their own fish fillet by following the handout picture instructions. After the child had felt the fillet with his/her fingers to ensure that no fish bones were present, the fillet was ready to be cooked.

Phase c: Cooking: The fish fillets were turned in rye flour containing salt and pepper and were then fried in butter on a hot pan. This is the traditional way of cooking fish fillets in Danish cuisine.

Phase d: Tasting: The fried fish fillets were served on a slice of rye bread with butter and a slice of lemon. Tasting/eating was voluntary. This is a common way of serving fish fillets in Danish cuisine. 

After the experiment, the children could take the gyotaku home, or the school could use it, for example in an art exhibition.

### 2.4. Data Collection—Participant Observation 

The participant observation was primarily concept-driven [55] and based on the framework of Rozin and Fallon’s [23] and Rozin and Vollmecke’s [27] taxonomy of food rejection and acceptance. Therefore, a loosely structured observation guide, with room for exploratory inquiry, was constructed based on the main framework of acceptance and rejection with the following themes: (1) the social/group interaction element, (2) the children’s interaction with the fish, (3) the process of the exercise, and (4) development/changes in attitude throughout the experiment. Documentation methods used during the participant observation were in the form of written field notes and situational photos to document the setting, various situations, and child–fish interactions. The field note strategy was inscription and transcription [55], in which descriptions of behaviours (inscriptions) and informants’ own words and dialogues (transcription) were recorded in an observational journal based on the loosely constructed and pre-thematised observational guide.

The same researcher participated in all gyotaku exercises by observing and interacting with the children through informal conversations based on the observation guide. In all cases except two (school MB and MD), observation assistants were present throughout the gyotaku exercise. At schools SA, SB, SC, three observation assistants were present, and at school MC one observation assistant was present. In all cases, the observation assistants had a semi-participatory role while also documenting the gyotaku exercise through photos. During the participant observations, researchers and assistants interacted with the children through informal conversations based on the situation while the children were working with the fish. Questions were based on “free narrative” [56] to promote situational comfort and to get and keep the conversation flowing. The questions were directed towards the children’s perspectives of the situational experiences; for example (to the whole group): “How is it going here?” and “How do you feel about filleting a fish?”. Probing [53] was used to follow up on short answers, for example “Can you tell me some more about that?”. The focus was on informality and conversations steered by the children and their point of view. If a child asked what had been written down during a conversation, he/she was given the opportunity to read it. After each observation session, observational journals and photos were compared and evaluated. Post-intervention notes were documented by the research group. Furthermore, the field notes in the observational journal were immediately after the observation separated into direct observations of behaviour, dialogues based on children’s peer-to-peer dialogues and researcher–child dialogues, and researcher reflections. A pre-coding was conducted based on concept-driven coding [55]; for example based on the framework of acceptance and rejection [25,27], fish handling, sensory aspects, and group work.

### 2.5. Data Analytical Method

Data analysis was conducted by using Applied Thematic Analysis (ATA) developed by Guest, MacQueen, and Namey [57]. ATA was applied to identify themes and to analyse patterns of meaning in relation to the research questions under study and was chosen for its flexibility with regard to type of texts, for example field notes [57], and its ability to highlight similarities and differences across cases [58].

**Figure 3:**
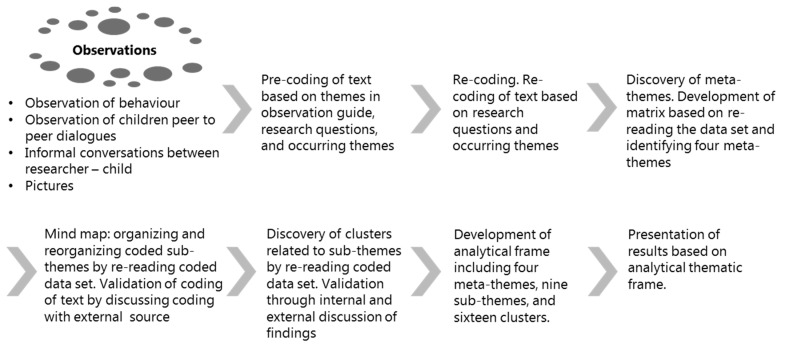
The ATA (Applied Thematic Analysis) data processing.

**Figure 4:**
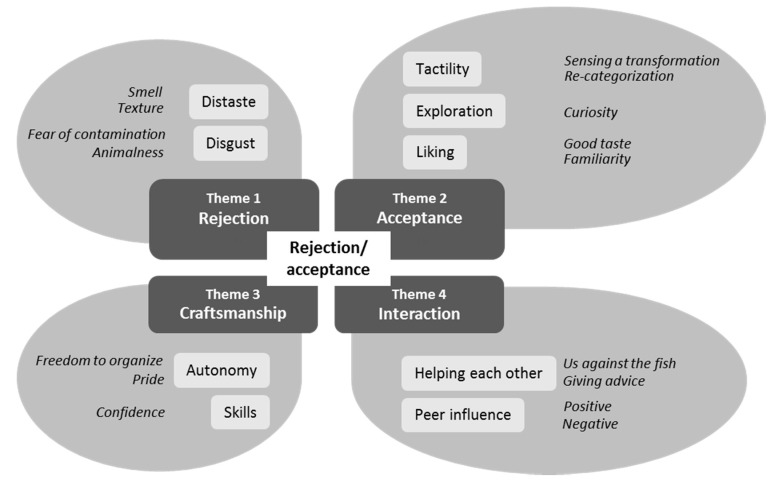
ATA frame for presentation of data: main category, meta-themes, sub-themes, and related clusters.

Through a concept-driven [55] processing of data based on the research questions, four meta-themes were identified by organizing the pre-coded text into a matrix based on the frequency of re-occurrence of documented observed behaviours and dialogues. The identified meta-themes were rejection, acceptance, craftsmanship, and interaction. A thematic map was constructed to create a visual outline of possible sub-themes [57,58]. Finally, themes were re-considered to ensure accurate representation by re-reading the data set [57,58]. (See Figure 3 for presentation of the ATA data processing. This resulted in the appearance of sub-theme clusters as situational events, behaviours, etc. (see Figure 4).

Data not relevant for the research questions were excluded from the data set and analysis after being re-read to ensure lack of relevance. Furthermore, the ATA frame (analysis, results, and discussion hereof) was read by and discussed with researchers within the research group, but for those who had not been present at the interventions the frame was read by and discussed with an experienced researcher outside of the research group.

The essence of meta-themes and sub-themes are presented in Table 2. Data were not only sorted by meta-theme and sub-theme but also by exercise phase (see Appendix Table A1: Data set).

## 3. Results

In Figure 4 two main categories, four meta-themes, nine related sub-themes, and sixteen clusters *(italic)* are presented. 

Data are presented according to the ATA frame (Figure 4) by including relevant examples from the data set to support the ATA. Abbreviations applied in the analysis: Obs: observation, ic: informal conversation. Phases of the exercise: ^#1^ = Before printing; ^#2^ = During printing; ^#3^ = Between printing and filleting; ^#4^ = During filleting; ^#5^ = Frying; ^#6^ = Tasting.

### 3.1. Meta-Theme 1: Rejection

#### 3.1.1. Sub-Theme: Distaste

Rejection based on distaste, which includes all sensory characteristics, both real or imagined [25,26], was based on two main sensory characteristics: smell and texture. Rejection based on smell was primarily present in two phases of the experiment. Firstly, at the beginning of the printing phase when the children were presented with the fresh fish:

^#1^ When the lid is removed from the fish on ice, several children say: *“Ugh, it smells fishy”* [in a bad way] (School all, obs.)

Secondly, smell was a source of rejection based on distaste in the final experiment phase (tasting):

^#6^ Some children do not want to taste the fish. Int.: *“Why?”* Response: *“It smells of fish. We know we do not like fish because it feels weird in the mouth”*. A girl says: *“That is also why my dad does not like fish”* (School MC, ic).

Furthermore, the texture of the fish in the mouth was a factor in rejecting the fish based on distaste:

^#6^ A girl nibbles on the fried fish: *“I don’t like the fish. It is kind of… mushy”*. (School MD, obs).

^#6^ Everyone in the class tastes the fried fish, but three boys spit it out and agree that they do not like to chew it as it is too mushy and soft in the mouth. (School MB, obs).

#### 3.1.2. Sub-Theme: Disgust

Apart from behaviours and verbal expressions promoting rejection based on distaste, rejection was also observed for the affective response of disgust. 

**Figure 5:**
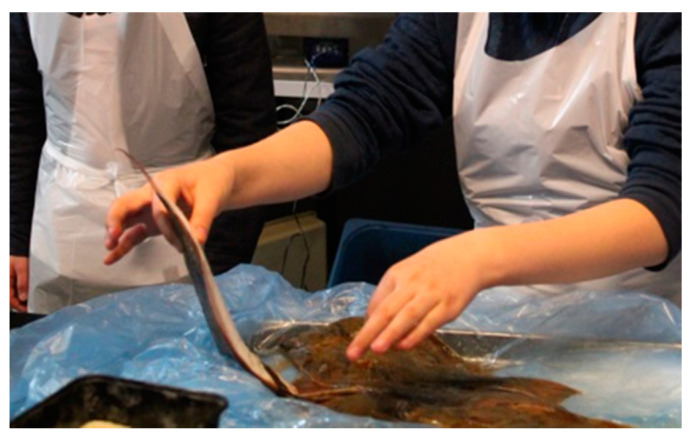
A display of disgust: picking up the fish, photo: R. Højer.

Fear of contamination was observed primarily in two situations. Firstly, at the beginning of the experiment (phase a) when children picked up the fresh fish using only the tips of their thumb and index finger as shown in Figure 5. Most often the task of picking up the fish would be done by two children going to the fish box. One would pick up the fish (as illustrated in Figure 5) while the child not picking up the fish would often stand in the background in order to not get too close to the fish, although still leaning forward to have a look.

Secondly, in relation to filleting (phase b):

^#4^ Several children put on latex gloves before starting filleting (School MB, obs.).

Rejections driven by disgust also appeared as a reaction to the idea of “animalness”. These reactions were also predominant at the beginning of the experiment (phase a) and during the filleting phase (phase b):

^#1^ Girl, when fish has been collected: “*Yuck! Look, it has eyes”* [pinches her nose] (School SC, obs).

^#4^ Int.: *How is it going with filleting the fish?* The girl cutting responds: *“I think that sound when you kind of hit the bone with the knife and that sound it makes…ugh”* [shrugs] (School MD, ic).

^#4^ Girl, during filleting: “*Yuck, it has fish guts inside* [viscera]” [she pinches her nose and turns away, holding her hands in front of her mouth] (School MD, obs).

### 3.2. Meta-Theme 2: Acceptance

#### 3.2.1. Sub-Theme: Tactility

Acceptance through tactility was observed in two forms: “sensing a transformation” and “reduction of animalness” through the sense of touch and a re-categorisation of the fish from animal to non-animal. The former displayed itself at the beginning of the experiment (phase a) after the fish’s natural mucus layer had been washed and removed:

^#1^ Boy group after washing the fish: they stroke it and agree that it is weird because it was so slimy before but now it is soft to the touch (School MD, obs).

When the children started the printing process (phase a), it seemed like the fish had been re-categorised from animal to an art medium. Touching the fish was no longer an issue:

^#2^ During the printing process, great attention is given to getting the right amount of ink on the eyes, fins, and the mouth to get them onto the paper. This is done by unfolding the fins with their fingers and dabbing the sponge lightly on the eyes, fins, and the mouth (School all, obs.).

^#2^ Between prints, the fish is gently patted and stroked by several children; it is “tickled” between the eyes and around the mouth (School all, obs).

#### 3.2.2. Sub-Theme: Exploration

Exploration was predominant in two main scenarios: exploring the fish before and after filleting (phase b). There were clear signs of curiosity, as shown in the following example:

^#3^ A girl is exploring the fish. She opens the fish’s mouth and looks into it: *“I just had to look inside. You can see its teeth…I just had to touch”*. Another girl in the group: *“Ohh yes, its mouth can get really big”*. The first girl replies: *“Yes, it can eat big fish”* (School MC, ic).

**Figure 6:**
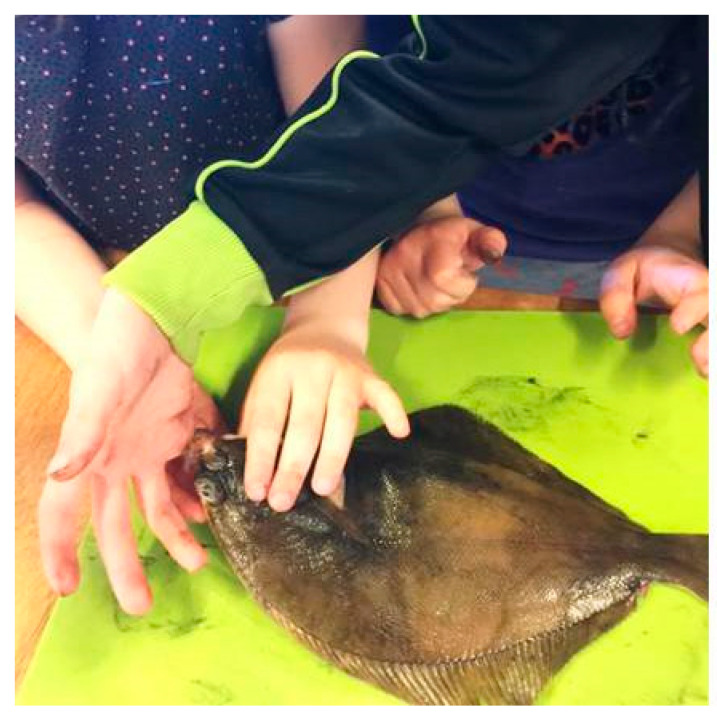
Children exploring the fish, photo: R. Højer.

This exploratory scenario is also seen in Figure 6 with children putting their fingers in the fish’s mouth to feel its teeth.

After filleting, children explored the fish:

^#4^ Roe in fish: at first the children do not want to touch or even look, but after a while they start to pick at it with the knife tip, and then cut it, mash it, and study the small eggs (School SA, SB, SC, obs.).

Both exploratory scenarios led to a greater child interaction with the fish.

#### 3.2.3. Sub-Theme: Liking

Acceptance due to liking was primarily driven by the sensory characteristic “taste” (the fish tasted good). It also seemed like taste familiarity was a factor in liking it.

^#6^ A girl is eating her fish fillet: *“Mmm, I love fish fillet”* Int.: *“Why?”* Girl: *“It is kind of a little bit sweet but also just good. We also get it at home”* (School MD, ic).

^#6^ A girl tastes a little bit of roasted fish roe and says: *“Mmm, it actually tastes like cod roe… but it is a little bit grainy and dry in the mouth”* (School MD, obs).

### 3.3. Meta-Theme 3: Craftsmanship

#### 3.3.1. Sub-Theme: Autonomy

Throughout the experiment, autonomy was a sub-theme, since all of the assignments were carried out through group negotiation and decision-making; there was freedom to organise the work themselves (no teacher involvement), for example, who should pick up the fish, who should fry the fish etc. Pride in their work was especially evident during printing (phase a) and filleting (phase b):

^#3^ After the printing, children show their self-made print to teachers and other groups (School all, obs).

^#4^ They want to try to fillet the fish themselves. The experimenter (first author) is not allowed to help too much, only to correct them if they have made a wrong cut (School all, obs).

#### 3.3.2. Sub-Theme: Skills

Skills were developed, particularly in the filleting process (phase b). It was observed that the children initially had difficulties in holding the knife correctly and actually filleting the fish. During the filleting process, they became more confident in using the knife and in how to fillet the fish (School all, obs.). During cooking (phase c), skills were developed when they were trained how to cook a fish for the correct amount of time:

^#5^ While frying the fish, the children are very preoccupied with cooking it for the right amount of time so it is not raw, but they are also focused on not cooking it for too long. They comment on the colour and use it as a way of telling if it is done (School all, obs).

A clear indication of the acquired skills can be seen in the following extract:

^#5^ After frying the fish, a girl says: *“Ah, now I know how to make fish fillet. I would like to try it at home if mom will buy a fish”* (School MC, ic).

### 3.4. Meta-Theme 4: Interaction

#### 3.4.1. Sub-Theme: Helping Each Other

The sub-theme “helping each other” appeared primarily as “us against them/the fish” and giving advice. A concept of “we are in this together” and “us against the fish” appeared, particularly at the beginning of the experiment (phase a), where the children had to pick up the fish and prepare it for printing:

^#1^ Two girls are washing and drying a dab before printing. They help each other by holding the fish at each end and carrying it together to the printing table (School MC, obs.).

Children also helped each other when washing the fish to remove the fish skin mucus prior to the printing (phase a). For example, one child supported the fish’s tail, while another rubbed it with a lemon slice to remove mucus from the fish. Furthermore, helping each other was observed when, for example, applying the ink and giving advice on how to apply ink to the fish during printing, and giving advice on how to make a correct cut with the knife during the filleting phase (phase b):

^#4^ The girls give advice on how and where to cut: *“You have to start with the moon-shaped cut there”*. The boys correct each other more often (School MB, obs).

#### 3.4.2. Sub-Theme: Peer Influence

Peer influence was observed throughout the exercise and resulted in the other children in the group reacting either positively or negatively to the fish. The following two extracts illustrate peer influence leading to a positive reaction to the fish (the first extract) and a negative reaction to the fish (the second extract):

^#3^ After printing, two girls in a group of four are touching the fish, while the other two do not want to touch it. After observing the girls touching the fish for a little while, the other two girls change their mind and come over to the fish and try to touch it (School MB, obs.).

^#6^ Everyone in the class tastes the fried fish, but three boys from the same group spit it out and agree that they do not like to chew it as it is too mushy and soft in the mouth (first one boy spits it out, then the rest of the group) (School MB, obs).

**Figure 7:**
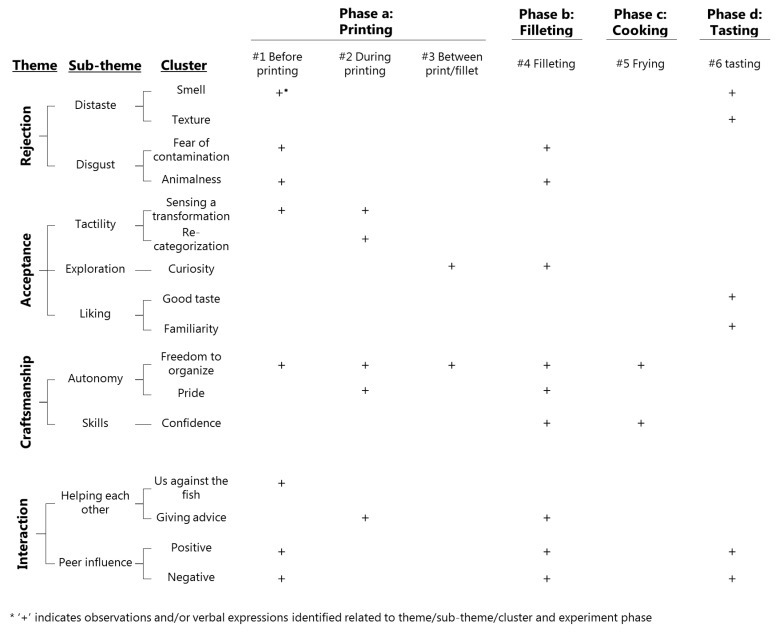
Applied thematic analysis (ATA) summary visualised.

The ATA is summarised visually in Figure 7, which shows meta-themes, sub-themes, and predominant clusters within identified sub-themes related to the different phases in the experiment.

## 4. Discussion

**Figure 8:**
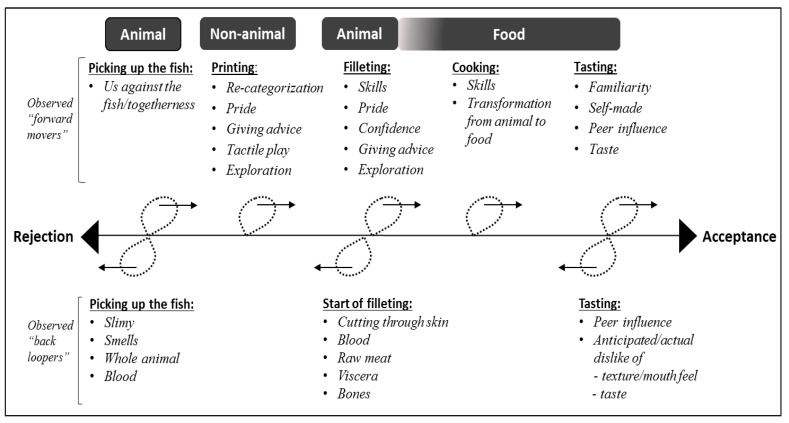
A rejection–acceptance continuum based on fish categorisation with examples of elements driving acceptance forward or backwards. Developed by first author Højer, inspired by and Rozin and Fallon [24].

Based on thematic analysis, we propose the following diagram to explain a rejection–acceptance continuum (Figure 8). 

Figure 8 illustrates elements which drive either rejection or acceptance along the continuum. Our observations on the categorisation and re-categorisation of the fish as animal, non-animal, and food were in line with what Rozin and Fallon [63] and Martins and Pliner [25] refer to as “animalness”. This animalness can be reduced in a fish by, for example, removing the head and bones, cutting it up, cooking it, and serving it without it resembling what it is: a fish and an animal [63].

At the beginning of the exercise, the fish was categorised as an animal due to its smell, slimy texture, and visual appearance (whole animal with head, fins, blood, etc.) and thereby promoted rejection. The whole fish represented a high degree of animalness, since it did not resemble what the children would typically eat (a breaded fish fillet). According to the results of a Danish citizen science project on Danes’ fish eating habits, the hot fish dish most often eaten by children was breaded fish fillet (39%), and in the form of cold cuts (eaten on rye bread) the favourites were mackerel in tomato sauce (33%) and fish cakes (21%) [64]. According to Fischler [65], this could be categorized as gastro-anomie, because the consumer has problems in identifying food and food origin as a result of processing [65]. Rejection based on animalness was also found in a Norwegian study on adolescents’ (16 to 17 years of age) attitudes towards meat from farm animals. Females, in particular, rejected meat due to its association with, for example, blood and animal parts [66]. The study also found that participants in regular contact with farm animals displayed no disgust reaction and had a more relaxed attitude towards meat production [66]. In an empirical study on what motivates food disgust, Martins and Pliner [25] found that animalness was not the complete explanation for a food disgust reaction, as non-animal food products were also capable of promoting disgust. According to Martins and Pliner [25], an explanation could be found in the experienced texture of, for example, slime, as it could be related to decay. Through multidimensional scaling analysis, they were able to identify independent (i.e., unique) dimensions, suggesting that both aversive textural properties and the reminders of animalness are primary variables accounting for perceptions of food disgust [25,67]. Egolf, Siegrist, and Hartmann [28] also found in their study on how people’s food disgust sensitivity shapes eating and food behaviour that surface texture of food was capable of promoting disgust. 

According to our observations during the printing phase, the fish was re-categorised from animal to non-animal, because it was perceived as an art/play medium and rejection cues were not evident. In this phase, tactility through touching the fish, as part of the assignment of printing, appeared to promote acceptance of the fish. This observation correlates with the findings of Coulthard and Sealy [38], who found that pre-school children tried more fruits and vegetables after participating in a sensory play activity with real fruits and vegetables than children in a non-food sensory play task (*p* < 0.001) and in a visual exposure task (*p* < 0.001). Similar results were also found by Nederkoorn, Theiβen, Tummers, and Roefs [41]: tactility increased the acceptance of food with the same texture.

The observed acceptance could also be promoted by the reduction in mucus on the fish after washing, which would reduce the texture-induced disgust as proposed by Martins and Pliner [25]. Nevertheless, this does not account for the following tactile exploration of the fish, where the children, driven by curiosity about something unfamiliar, put their fingers in the fish’s mouth, touched the gills, eyes, tongue, etc., with all parts of the fish still covered by or containing mucus (see Figure 6).

At the beginning of the filleting phase of the exercise, the fish was again categorised as an animal, and rejection was promoted. A behavioural example was the observation of the children putting on latex gloves in this phase, although this behaviour could also be a result of peer influence. 

Rejection was primarily due to the cutting through of the skin of the fish and cutting close to the bones. Both sound and visual cues reminded them that they were cutting into an animal, thereby increasing the perceived animalness. Later in the filleting phase, the fish were re-categorised from animal to food, because the fish was now fish fillets. The bones, skin, viscera, head, etc. were disposed of. What remained was a form of the fish that was familiar to the children: fish fillets. Applying Lévi-Strauss’ [68] concept of nature-culture, we see that, through the filleting process, the fish had gone from a natural form to a more cultivated form, and through the frying of the fish the final step in the cultivating process had been reached. 

Furthermore, during the filleting and cooking phase, the children started to learn technical skills, and they clearly took pride in their work, which could be an expression of what Sennett [59] calls the emotional reward for attaining a skill and doing it well, like a craftsman. This finding of promoted self-efficacy, as defined by Bandura [69], is supported by the findings of Cunningham-Sabo and Lohse [70] in an interventional study with fourth-graders. Not only did they find an increase in cooking and food self-efficacy but also an increase in fruit and vegetable preference. Most notable is the finding that non-cookers particularly benefitted from the intervention [70]. An increase in cooking efficacy was also confirmed in a similar study including an experiential approach by Jarpe-Ratner, Folkens, Sharma, Daro, and Edens [71], although no definition of the concept experiential was given.

At the end of the experiment, the fish were fried, and the re-categorisation from animal to food was complete. Observations showed that the majority of children chose to taste and eat the fish fillet on rye bread; the reason given was the good taste, a reason corresponding to the findings of Sick, Højer, and Olsen [29] in a study on children’s self-evaluated reasons for accepting and rejecting foods.

Rejection was promoted by, for example, the texture of the cooked fish in the mouth. Rejection of fish based on texture was found by Donadini, Fumi, and Porretta [72], where fish was rejected due to softness, a jelly-like texture, fast melting, and tendency to fall apart easily textures. Texture was also found to be a key rejection characteristic by Sick, Højer, and Olsen [29]. For the children that showed reluctance throughout the experiment and ended up tasting the fish fillet, an “I filleted and cooked it myself” effect could be a possible explanation. A similar effect of “I cooked it myself” was found by Dohle, Rall, and Siegrist [73] and Allirot, da Quinta, Chokupermal, and Urdaneta [74]. Allirot et al. [74] and van der Horst, Ferrage, and Rytz [75] also point to the context or atmosphere in which the food exposure took place and the “cooking together” factor as relevant factors impacting food likes and dislikes and thereby promoting acceptance or rejection. Since the gyotaku experiment took place in a school(-like) setting, the “cooking together” and “helping each other” factors promoted acceptance of fish. According to Lukas and Cunningham-Sabo [76], there is a difference between cooking with friends and classmates. In a qualitative study, they found that classmates were typically associated with rules, structure, and restrictions, while friends are defined by fun and freedom [76]. Yet, when Lukas and Cunningham-Sabo [76] compared data across focus groups, they found that the cooking and tasting group did not make a clear distinction between classmates and friends, and the children in this group seemed to consider their classmates as friends in this “cooking together” context. This was not the case in the two other groups. However, other studies [77,78] have not found a correlation between an experience-based approach and positive change in acceptance, preference or liking of foods.

## 5. Strengths and Limitations 

This section considers the credibility, transferability, dependability, and confirmability [53,79] of the study findings.

Credibility was sought in this study by comparing the findings with those of previous studies that have focused on similar research. Furthermore, to reduce observer bias, observer assistants were present in all but two cases, and after the experiment had ended, dialogues took place between the experimenter and assistant regarding what had been observed. Dependability was sought via a thorough description of the study design and the gyotaku experiment itself in order to ensure that other researchers are able to execute a study in a similar way. Even though true objectivity of the researcher rarely exists, confirmability was sought through a sampling process, whereby the participating classes entered the study according to the rule of “first responders to the information letter” sent out via email (regional) and shared in a Food-Knowledge-specific Facebook group for teachers (national). 

Geographically, the data were only collected in the eastern part of Denmark. Therefore, in terms of transferability, it could be said that this case study is not representative of the general population of children in Denmark. Nevertheless, the findings can be seen as indications transferable to similar contexts, since the observations seemed stable and comparable across the cases (schools and classes). Furthermore, even though the experiment varied in terms of setting, the observations across the two settings seemed stable and comparable. Additionally, a research participation effect [80] cannot be completely eliminated as the presence of the research and assistant group in the gyotaku experiment situation is an addition to the typical setting situation. Furthermore, analysis and ATA frame was validated by a researcher not part of the study, but with extensive research experience to reduce bias.

Even though more research in this area is needed, this finding opens up the possibility of transferring the gyotaku exercise and the expected outcome to settings outside the conventional school setting.

We recognise that the present study holds certain limitations investigation-wise that need to be addressed in future research. One such limitation is the aspect of how children categorise and re-categorise fish and how this is connected to the experimental context, the school arena. Furthermore, a more focused investigation of how tactile play might influence children’s acceptance of food, especially outside the area of fruit and vegetables, is warranted.

## 6. Conclusions

With regard to how children responded to handling, preparing, and cooking the fish and how the process of the gyotaku experiment affected the acceptance, we identified that response of rejection and acceptance moved back and forth on a continuum. Rejection was driven by slimy touch, whole animal, smell, cutting through skin, texture of fish meat in the mouth, and taste, and acceptance was promoted by togetherness, helping each other, tactile play, re-categorisation of the fish, exploration, pride, skills, and was self-made. Furthermore, the movement back and forth was determined by how the fish was categorised (as animal, non-animal, or food). The study revealed that autonomy, skills, pride, and helping each other in the groups were important factors in promoting acceptance, whereas the texture of the fish, for example, led to rejection. Furthermore, we found that using the fish as a creative medium for tactile play became an important motivator in promoting acceptance. The findings in this study highlight that cooking combined with tactile play could be a way of promoting acceptance of fish, and as such serve as a potential strategy in promoting healthy food behaviour. The same exercise could be used with other food groups as well, for example with vegetables, fruit, chicken (e.g., print of feet or wings before preparing) etc. where the squid ink is substituted with berry juice or beet root juice. At the same time, our findings support the importance of the school setting and the subject Food Knowledge as a potential experiential learning gateway to promoting healthy food behaviour through focusing on children’s food and culinary knowledge and skills, which has also been recommended by Nelson et al. [47].

## Figures and Tables

**Table 1:** Participant characteristics.

School	Classes	Grade	*n*	Sex (♀/♂)	Teachers *
School SA **	1	6th	32	21/11	2
School SB	1	6th	24	14/10	2
School SC	1	5th	18	10/8	2
School MB ***	1	6th	21	13/8	1
School MC	1	5th	18	9/9	1
School MD	1	6th	19	11/8	1
Total	6		132	78/54	9

* Number of teachers present during the gyotaku exercise. ** Schools SA, SB, and SC are schools from workshops during Science Week 2016. *** Schools MB, MC, and MD are schools from the main study 2017.

**Table 2:** Essence of meta-themes and sub-themes.

Meta-Theme/Sub-Theme	Essence
1. Rejection: distaste and disgust	The theme ‘rejection’ concerns children’s behaviour and verbal expressions that can be characterised as distaste or disgust as defined by Martins and Pliner [25], Rozin and Fallon [24], and Angyal [35]; distaste is defined as a sensory-driven reaction (e.g., smell, touch, taste, appearance, texture, sound), and disgust as a concern with contamination or being soiled as a result of contact with what is perceived as animal bodily waste products. The latter is defined by observed body language, for example turning away, holding a hand in front of the mouth and/or nose, mimicking nausea and/or vomiting, etc. [24,35]. The theme refers to observed behaviour and verbal expressions motivated by any interaction with the fresh fish, which could promote or is a direct rejection of tasting the cooked fish at the end of the experiment. Rejection could also be a result of a perception of a food [27].
2. Acceptance: tactility, exploration, and liking	The theme “acceptance” concerns children’s behaviour and verbal expressions concerning tactility, limited to include the sense of touch with the hands, exploration driven by curiosity, and liking, which refers to a positive affective response to food. Acceptance is understood as a willingness to taste the food, but it can then be rejected. Acceptance does not depend on liking, since other motives can exist for accepting a food (e.g., for its health benefits) [24,27].
3. Craftsmanship: autonomy and skills	The theme “craftsmanship” concerns observed behaviour and verbal expressions related to the defined activity of preparation and cooking. Craftsmanship is understood as a physical, bodily practice that leads to a tactile experience and relational understanding [59]. Craftsmanship covers a tacit experience-based set of knowledge and skills within cooking – in this case, the fish. Even though Sennett [59] defines craftsmanship as “the skill of making things well”, in this case the effort and attempt matter just as much, and maybe more than the outcome, an approach also supported by Martin [60]. In craftsmanship, Martin [60] underlines the importance of creating an environment in which the child feels independent and thereby learns by making decisions. In this space of autonomy, intrinsic motivation may promote pride in the work, thereby increasing curiosity with regard to tasting the fish.
4. Interaction: helping each other and peer influence	The theme “interaction” refers to observed behaviour and verbal expressions related to social facilitation either related to the children helping each other or by peer influence. Through behaviour and verbal expressions, the children might influence each other with regard to accepting or rejecting the fish at the end of the experiment [27,61,62].

## Appendix A

**Table A1:** Data set: Meta-themes, sub-themes, and data extracts from field note journal (obs: observation *, ic: informal conversation **).

Meta-Theme	Sub-Theme	Data Extract
1. **Rejection**	**Distaste**	^#1^. When the lid is removed from the fish on ice, several children say “Ugh, it smells fishy” [in a bad way]. (School MB, MC, MD, SA, SB, SC, obs *).
		^#6^ Some children do not want to taste the fish.
		Int.: *“Why?”*
		Response: *“It smells of fish”, “We know we do not like fish because it feels weird in the mouth”*. A girl says: *“That is also why my dad does not like fish”* (School MC, ic**).
		^#6^ Int.: “*Do you like the fish?”* (asked to a girl group after frying the fish). Girl, not eating her fish fillet: *“I do not like the smell of fish”*. (School SB, ic).
		^#6^ After tasting the fried fish fillet, a boy said: *“Arhh, that is not for me”.*
		Int.: *“How come?”*
		Boy: *“It feels mushy in my mouth and tastes fishy”.* (School SA, ic).
		^#6^ Two girls absolutely do not want to taste the fried fish, because they know that they do not like fish. (School SB, ic).
		^#6^ A girl nibbles at the fried fish: *“I don’t like the fish. It is kind of… mushy”*. (School MD, obs).
		^#6^ Three boys did not want to taste the fish: *“We do not like the taste and smell of fish”*. (School SC, ic).
		^#6^ Everyone in the class tastes the fried fish, but three boys spit it out and agree that they do not like to chew it as it is too mushy and soft in the mouth. (School MB, obs).
		^#1^ When the lid is removed from the fresh fish, many children react by turning away from the fish, holding their hands in front of their mouth and/or nose, pinching their nose, mimicking vomiting, making “yuck” noises, closing their eyes, etc. (School MB, MC, MD, SA, SB, SC, obs).
		^#1^ *“Ugh, it is GROSS and soooo slimy…”*
		Some children mimic vomiting (School SA, obs **.).
		^#1^ Girl, after fish has been cleaned and is placed on the cutting board: *“It is not normal”*. (School MD, obs.)
		^#1^ Girl, when fish has been collected: “*Yuck! Look, it has eyes”* [pinches her nose]. (School SC, obs).
		^#1^ A boy does not want to touch the fish: *“It is slimy”*. [no special facial expression/body language]. (School MD, obs).
		^#1^ A boy pokes the fish before washing: *“Ugh, it is sticky”*. (School MB, obs.)
	**Disgust**	^#1^ Several children try to pick up the fish from the box using only the tips of their thumb and index finger (School MB, MC, MD, SA, SB, SC, obs).
		^#2^ A boy says that the fish is really disgusting, makes “yuck” sounds, but at the same time he cannot help himself poking it in the eye followed by big arm swings and screeching. Then he runs over to wash his fingers and goes back and pokes the fish again. (School MD, obs).
		^#4^ A group of girls purse their lips at the sight of blood from the fish. Some close their eyes and turn away from the fish. (School MD, obs.).
		^#4^ Int.: *How is it going with filleting the fish?* (Question to a girl group).The girl cutting responds: “*I think that sound when you kind of hit the bone with the knife and that sound it makes…ugh”* [shrugs] (School MD, ic).
		^#4^ During filleting. Girl: “*Yuck, it has fish guts inside* [viscera]”. [she pinches her nose and turns away while holding her hands in front of her mouth]. (School MD, obs).
		^#4^ Several children put on latex gloves before starting filleting. (School MB, obs.).
		^#5^ When the fillets have to be turned in breadcrumbs, they are moved/lifted by holding the fillet in the tail end with the tip of the thumb and index finger (to touch as little meat as possible). (School MB, MC, MD, SA, SB, SC, obs.).
2. **Acceptance**	**Tactility**	^#1^ After washing the fish. Girl, stroking the fish: *“It is kind of rough but now it is soft”.* (School MB, obs).
		^#1^ Boy group after washing the fish: they stroke it and agree that it is weird because it was so slimy before but is now soft to the touch (School MD, obs).
		^#1^ Int.: *“What was it like to touch the fish?”*
		Girl: *“It was fun because when you stroke it in the opposite direction, it was… kind of rough”*. (School MC, ic).
		^#2^ During the printing process, great attention is given to getting the right amount of ink on the eyes, fins and the mouth to get them onto the paper. This is done by unfolding the fins with the fingers and dabbing the sponge lightly on the eyes, fins and the mouth (the girls are more aware of this than the boys). (School SA, SB, SC, MB, MC, and MD, obs.).
		^#2^ Girl: *“Use your fingers, it’s much easier”*.
		The group quickly shifts from using a spoon to using their fingers to ensure that the paper absorbs ink during the printing [stroking the fish on top of the paper]. (School MD, obs.).
		^#2^ Between prints, the fish is gently patted and stroked by several children; it is ‘tickled’ between the eyes and around the mouth. (School MB, MC, MD, SA, SB, SC, obs).
		^#3^ A girl group are stroking their fish and give it a name (School SA, obs).
		^#3^ A girl group are gently stroking their fish, and a girl says: *“I can’t eat it now”* (School SB, obs.).
		^#4^ After the filleting process, they use their fingers to check for small bones in the fillets (School SA, SB, SC, MB, MC, and MD, obs.).
	**Exploration**	^#2^ A boy turns the fish to its white side and asks: *“Why is it white underneath?”* Int.: [gives an explanation]. Boy: *“Ohh, that is smart”*. (School MC, ic).
		^#3^ A girl is exploring the fish. She opens the fish’s mouth and looks into it: *“I just had to look inside. You can see its teeth…I just had to touch”*.
		Another girl in the group: *“Ohh yes, its mouth can get really big”*.
		The first girl replies: *“Yes, it can eat big fish”*. (School MC, ic).
		^#3^ The children open the mouth of the fish and feel inside with their fingers. Feeling the teeth, in particular, makes them more curious, and they keep exploring, also by touching the tongue. (School SA, SB, SC, MB, MC, and MD, obs.).
		^#3^ Girl: *“Can you eat the squid ink?”*
		Int. *“Yes, you can. Do you want to taste it?”*
		More children gather around the table, and several of them taste the ink.
		*“Ugh, it is very salty”*. (School MC, ic).
		^#3^ After printing, a boy asks: *“Can you eat the eyes… and may I?”* (School MB, obs).
		^#3^ Int.: *“Have you ever tried to open the mouth of a fish?”*
		Boy group: *“Nooo…”.* Int.: *“Try it”.* A boy holds the fish, while another boy opens the mouth. All: *“Whoa!”*. (School MB, ic).
		^#4^ A girl says: “*The viscera are not disgusting but mysterious”.* (School MD, obs).
		^#4^ Boys start to explore the viscera of the fish. They ask what parts they are and whether they can be eaten. (School MB, MC, MD, SA, SB, SC, obs).
		^#4^ Boys start to pull out the intestines in their full length. (School SA, SC, obs.)
		^#4^ Girls cutting roe out from the fish.
		Int.: *“Do you know what that is?”*
		Girl: *“No…”*
		Other girl in group: *“I do… it is roe. Can you eat it?”*
		Int.: *“Yes”*
		Girl: *“Let’s try and fry it and taste it”.* (School SC, ic).
		^#4^ Roe in fish: first the children do not want to touch or even look, but after a while they start to pick at it with the knife tip and then cut it, mash it and study the small eggs. (School SA, SB, SC, obs.).
	**Liking**	^#1^ Girl: *“It smells good and bad at the same time”* (School MD, obs.).
		^#1^ Girl, when the lid is removed from the fish: *“It smells fresh… of the sea and salt”*. (School MB, obs).
		^#6^ A girl who says that she does not like fish chooses to taste it anyway: *“Ohh, but it tastes like chicken”.* (School MB, obs.).
		^#6^ A girl eats fried roe: *“Ohh, it tastes OK*—*just like the rest of the fish”*. (School SB, ic).
		^#6^ A boy fries the liver: *“It tastes like chicken—not bad… like chicken and a little bit of blood”.* (School SB, obs).
		^#6^ A girl tastes a little bit of roasted fish roe and says: *“Mmm, it actually tastes like cod roe… but it is a little bit grainy and dry in the mouth”*. (School MD, obs).
		^#6^. After the fish has been fried, a group of boys are talking about the taste of the fish. Boy: *“It actually tastes good”.* Another boy replies: *“Yes, much better than the ones I get at home”*. (School MC, ic).
		^#6^ Four boys taste the fried fish: *“Yes, it is good”*. The other boys agree by nodding their heads. (School MD, obs.).
		^#6^ A girl is eating her fish fillet: *“Mmm, I love fish fillet”*
		Int.: *“Why?”*
		Girl: *“It is kind of a little bit sweet but also just good. We also get it at home”*. (School MD, ic).
		^#6^ Most children choose to taste the fried fish. Only a few do not eat all of it (School SA, SB, SC, obs).
3. **Craftsman- ship**	**Autonomy**	^#all^ All assignments are carried out through group decision making and negotiation in the group (no teacher involvement), for example, who should pick up the fish, or who should fry the fish. (School MB, MC, MD, SA, SB, SC, obs).
		^#2^ A teacher wants to help a group with the printing, but the group says that they want to do it themselves. (School MC, obs).
		^#3^ After the printing, children show their self-made print to teachers and other groups. (School MB, MC, MD, SA, SB, SC, obs).
		^#4^ They want to try to fillet the fish themselves. I (the experimenter) am not allowed to help too much, only to correct them if they have made a wrong cut. (School MB, MC, MD, SA, SB, SC, obs).
		^#4^ A group asks for help with the filleting process, but the child holding the knife does not want to let it go (School MD, obs).
		^#4^ All of the children who filleted their own fish take great pride in their work; they show me their fillet and want me to praise them (prior to the filleting I made it clear that it was difficult and nobody can do it perfectly the first time they try it). (School MB, MC, MD, SA, SB, SC, obs.).
	**Skills**	^#1^ Before printing, groups evaluate the freshness of the fish based on what they remember from the theme course material (they remember the video material better than that from the booklet). They evaluate the freshness by smelling and agree that the fish should smell of salt and seaweed. (School MC, obs).
		^#2^ During the printing process, great attention is given to applying the right amount of ink to the fish and getting ink on all parts of the fish—this is more pronounced among the girls than the boys, who are more concerned with getting it done; a lot of them call me to show me their work. (School MB, MC, MD, SA, SB, SC, obs).
		^#4^ While filleting, several children refer to the You Tube video on filleting flatfish (a part of the theme course material): *“You just have to let the knife do the work for you”* becomes a phrase they repeat in the groups. (School MB, MC, MD, obs).
		^#4^ It is evident that the children are not used to filleting fresh fish; one class has been on a cooking camp where they worked with fish, but they did not try to fillet their own fish. (School MB, MC, MD, SA, SB, SC, obs/ic).
		^#4^ When the children start to fillet, they have great difficulty in holding the filleting knife correctly. However, when they try to fillet their own fish, they become more confident in using the knife and hold it more correctly. (School MB, MC, MD, SA, SB, SC, obs).
		^#5^ When frying the fish, the children are very preoccupied with cooking it for the right amount of time, so it is not raw, but they are also focused on not cooking it for too long. They comment on the colour and use that as a way of telling if it is done. (School MB, MC, MD, SA, SB, SC, obs).
		^#5^ After frying the fish, a girl says: *“Ahh, now I know how to make fish fillet. I would like to try it at home if mom will buy a fish”*. (School MC, ic).
4. **Child interaction**	**Helping each other**	^#all^ Groups are very preoccupied with justice; that all group members get to make a print, fillet and get to taste an equal amount of fish. (School MB, MC, MD, SA, SB, SC, obs).
		^#1^ Two girls are washing and drying a dab before printing. They help each other by holding the fish at each end and carrying it together to the printing table (School MC, obs.).
		^#1^ Two boys are collecting the fish from the box. They end up picking it up together and carry it to the sink. (School SA, obs).
		^#1^ A boy and a girl are helping each other, holding the fish and washing it under running water; one of them holds the fish, while the other rubs it with lemon. (School SB, obs).
		^#2^ During printing, the group members give advice to the child applying the ink, for example in order to get ink on the eyes, mouth and fins. Advice is also given to avoid large ink blobs on the finished print. (School MB, MC, MD, SA, SB, SC, obs).
		^#2^ During printing, they help each other apply the paper and place it correctly on the fish; they also help each other rub the paper and lift the fish print. (School MB, MC, MD, SA, SB, SC, obs).
		^#2^ The boys seem to correct each other, whereas the girls support each other (School MC, obs).
		^#4^. During the filleting, the group members give advice to the child filleting, for example, on how and where to cut. (School MB, MC, MD, SA, SB, SC, obs).
		^#4^ The girls give advice on how and where to cut: *“You have to start with the moon-shaped cut there”*. However, the boys correct each other more often. (School MB, obs).
		^#6^ Before eating, the children help arrange the fish fillets on small platters, so it looks like a small dish, while others set the table. They all sit down and eat at two tables laid with cutlery, glasses, water jugs and napkins. (School MD, obs).
	**Peer influence**	^#1^ When the lid is removed from the box containing fish, the disgust behaviour spreads in small groups—if one person in the group reacts, the others react too. (School MB, MC, MD, SA, SB, SC, obs).
		^#3^ After printing, two girls in a group of four touch the fish, while the other two do not want to touch it. After observing the girls touching the fish for a little while, the other two girls change their mind and come over to the fish and try to touch it (School MB, obs.)
		^#4^ While a group of boys explore the viscera and eyes of the fish, they challenge each other to touch the eye (School MC, obs).
		^#4^ During the filleting process, when children find viscera and roe in the fish, they start to react to it in the group. If one person reacts by holding a hand in front of the mouth, other group members react in a similar way. (School MB, MC, MD, SA, SB, SC, obs).
		^#4^ A girl does not want to fillet a fish, but after observing the other girls in her group, she ends up doing it (and even eating it after it has been fried). (School MB, obs).
		^#6^ Everyone in the class tastes the fried fish, but three boys from the same group spit it out and agree that they do not like to chew it as it is too mushy and soft in the mouth (first one boy spits it out, then the rest of the group). (School MB, obs).

Phase in the experiment: ^#1^ = Before printing; ^#2^ = During printing; ^#3^ = Between printing and filleting; ^#4^ = During filleting; ^#5^ = Frying; ^#6^ = Tasting; ^#all^ = All phases of the experiment. obs: observation *, ic: informal conversation **.
